# Prevalence and clinical characteristics of headache among medical students of Nepal: A cross-sectional study

**DOI:** 10.1371/journal.pone.0277821

**Published:** 2022-11-18

**Authors:** Ayush Mohan Bhattarai, Shekhar Gurung, Bishnu Deep Pathak, Saurab Karki, Ayush Adhikari, Om Prakash Tandon, Sabin Poudel, Deepak Yadav, Chiranjibi Pant, Bishal Dhakal

**Affiliations:** 1 Nepalese Army Institute of Health Sciences, College of Medicine, Kathmandu, Nepal; 2 Shivanagar Primary Health Care Center, Bharatpur, Chitwan, Nepal; 3 Military Hospital, Itahari, Nepal; 4 TU Teaching Hospital, Kathmandu, Nepal; 5 Lumbini Provincial Hospital, Butwal, Nepal; 6 Teku Hospital, Kathmandu, Nepal; 7 Shree Birendra Hospital, Kathmandu, Nepal; University of Eastern Finland: Ita-Suomen yliopisto, FINLAND

## Abstract

**Background:**

Headache is a common neurological disorder, with a global prevalence of around 50%. It may affect people of any age, gender, education, socioeconomic status and occupation. Tension headache, migraine headache and cluster headache are commonly encountered headache types. The prevalence of headache problems is higher among medical students. This could potentially affect their academic performance and quality of life. The objective of this study is to find out the prevalence of headaches and their clinical characteristics among students of a medical college.

**Materials and methods:**

An online, single-centre, cross-sectional study was conducted among undergraduate medical students in Nepal. Stratified sampling followed by a simple random sampling technique was adopted depending upon the academic years of students. For data collection, pre-tested semi-structured questionnaire was used. The data entry and analysis were done by using Statistical Package for the Social Sciences (IBM-SPSS), version-23. The prevalence of headache and its subtypes were calculated. All the clinical characteristics associated with headaches were also studied.

**Results:**

The prevalence of headache disorder was 65 (26.86%), with tension headache (69.23%) being the commonest one. It was highest among fourth-year students (37.84%) followed by first-year students (33.33%). Anxiety/stress (75.40%) was the most common precipitating factor. This problem stopped most of the students (63.09%) from doing daily activities. More than half of them (53.85%) practised self-medication in case of non-resolution of pain, and non-steroidal anti-inflammatory drugs (NSAIDs) were frequently used.

**Conclusion:**

Headache was fairly prevalent among medical students. Anxiety/stress in medical life has led to headaches in the majority of students. The headache disturbed their daily activities and promoted self-medication practice. So, this problem should be properly looked into and addressed in time by the concerned authority.

## Introduction

Headache is one of the most common neurological disorders with the estimated global prevalence in adults being around 50% [1]. It can affect people irrespective of age, sex, education, socioeconomic status and occupation [2]. Headache pain usually occurs due to peripheral nociceptors stimulation in response to tissue injury, damage or inappropriate activation of the pain-producing pathway of the central or peripheral nervous system, visceral distension, or other factors [3].

Broadly, all headaches fall under two subtypes: Primary headache and Secondary headache. Primary headache includes cluster, migraine and tension-type headache and secondary headache includes all those subtypes that result from underlying pathologies. A primary headache is, however, more common than a secondary headache [4].

The migraine headache is characterized by unilateral pulsatile or throbbing pain of moderate to severe intensity associated with nausea, vomiting, photophobia and phonophobia and lasting for about 4 to 72 hours if left untreated [5].

Similarly, tension-type headache is characterized by pain which is often described as “a band around the head”, pressing or tightening quality, mild to moderate in intensity, bilateral not affected by routine activity, and not associated with nausea and vomiting. Women are found to be at a greater risk to develop migraine and tension-type headaches. This could be because of the effect of female hormone levels, particularly estrogen [6].

Another subtype, cluster headache is defined by attacks of severe, unilateral pain which is orbital, supraorbital, temporal or in any combination of these sites, lasting 15–180 minutes and occurring from once every other day to eight times a day. The pain is associated with ipsilateral conjunctival injection, lacrimation, nasal congestion, forehead and facial sweating, rhinorrhea, ptosis and/or eyelid oedema, restlessness or agitation [4]. The lifetime prevalence of cluster headache is 124 per 100,000 and 1-year prevalence is 53 per 100,000. The ratio of episodic vs chronic cluster headache is 6.0 [7].

Medical students are among the vulnerable groups to headache disorder [8]. It is highly prevalent among undergraduate medical students. In past studies, stress was found to be the most common cause of headaches with sleep being reported as the commonest relieving factor. The academic performance, reasoning capacities, mental functioning and overall quality of life of medical students can be severely impaired due to headaches [9].

There are very few studies conducted on headache disorders in medical students in developing countries like ours. There has been studies in the developed nations which cannot be generalized to the developing nation such as ours. The medical students are subjected to a higher level of stress, performance pressure, longer duration of education and a strong responsibility towards their career. Neurological problems like headaches are commonly ignored in a developing country like Nepal by adults and also, they undergo self-medication. So, our main objective was to find out the prevalence of headaches and their different types among students of a medical college. We have also studied the possible severity, precipitating and relieving factors, and treatment practices among medical studies concerning headache problems.

## Material and methods

### Study setting

This study was conducted in a medical college in Nepal constituting a minimum of 100 students in every batch from Bachelor of Medicine and Bachelor of Surgery (MBBS) first year to the fifth year, representing people from different social and economic backgrounds. This medical college is located in the capital city, Kathmandu.

### Study design and participants

This was a cross-sectional study conducted among undergraduate medical students from the first year to final year at Nepalese Army Institute of Health Sciences (NAIHS)-College of Medicine, Kathmandu, Nepal.

### Sampling and sample size

Stratified random sampling followed by a simple random sampling technique was adopted.

Sample size:

Total population of MBBS students (N) = 540

Calculatedsamplesize(n)=(z^2pq)/E^2=3.8416*0.5*0.5/0.0025=385

Where,

E = margin of error, 5%
p = estimated proportion of the population which has attribute to the questions, taken as 50%
q = 1-p = 0.5
z = confidence interval at 95% = 1.96

Since the population was finite, the adjusted sample size was calculated as per Cochran’s formula [10]


$$\begin{array}{l} \text{Adjusted}\;\text{sample}\;\text{size}\;\left(\text{n}’\right)=\text{n}/\left[1+\{\left(\text{n}-1\right)\right)/\text{N}\}\\ \;=385/\left[1+\left(384/540\right)\right]\\ \;=385/1.71\\ \;=225 \end{array}$$

100% of the randomly selected participants responded to our questionnaires. However, while calculating the minimum sample size, we had assumed a 5% non-response rate. That made our sample size 236. Yet, we took 242 cases finally for our study.

After calculation of sample size, a stratified sampling technique was employed using students’ academic year as a stratum. First, the total sample size was proportionally allocated for each stratum (MBBS first year to the fifth year), and then a simple random sampling method using random number generator software was applied.

### Data collection and study variables

The data were collected using semi-structured questionnaires prepared by the investigators themselves after studying other similar studies and International Headache Society guidelines. The questionnaire was pre-tested in 10% of the sample before conducting the study, and necessary modifications were done to the questions. But we did not conduct any kind of reliability tests.

The final questionnaires were disseminated via online google forms. The list of medical students studying and their respective email addresses and contact numbers were obtained from the academic record section, and this was used as the sampling frame. An email containing a link to Google forms with the explained objectives was randomly sent to the selected students. A written consent form was attached along with the email. Single response from students was ensured via Google forms setting by choosing a “Limit to one response”. The participants who did not respond to the email were personally called by data collectors and were properly explained the objectives of the research and were requested to fill out the google form. Those students not willing to participate were planned to be omitted from the research. However, all the participants responded to our questionnaires following explanation and further request. Data was collected after the approval of IRC from 7^th^ February to 8^th^ March 2022.

The collected data included socio-demographic information like age, gender, education and marital status. Diagnosis of primary headache in the past was made by a neurologist or general physician after thorough clinical evaluations and investigations. There were no critical data collection procedures performed by the investigators. Some of our questionnaires were multiple choice (with provision of selecting more than one options) whereas others were open ended as well. The information was solely based on the participant’s reply. As per the participants with the diagnosis of primary headache, all baseline investigations and imaging were done to rule out secondary causes of headache. Based on the primary outcome, the cases were divided into tension headaches, migraine headaches, cluster headaches and any other types of headaches if there are. The secondary outcomes were characteristics of headache like the site, onset, the character of pain, any other associated symptoms, duration of headache, exacerbating or relieving factors, diurnal variation and impact of headache on quality of life. Any other warning signs before the headache began were also noted. Likewise, treatment or medication done for headaches was also noted.

### Ethical consideration

Ethical approval was taken from the Institutional Review Committee (IRC Reg. No. 520, Ref No. 245), Nepalese Army Institute of Health Sciences. The data was collected from students only after they consented to participate in the research. Consent was informed and written and was obtained from the questionnaire itself. The privacy and anonymity of patient information were well-maintained.

### Data analysis

The statistical analysis was done using Statistical Package for the Social Sciences (IBM-SPSS), version-23. The categorical variables were expressed in frequency and percentages and were presented appropriately in tables. The continuous variables were expressed in median and interquartile ranges (IQR).

The overall prevalence of headaches and their types was calculated. Likewise, its prevalence based on gender, marital status and academic years was also assessed. The p-value was obtained by performing the chi-square or Mann-Whitney U test, wherever necessary. A P-value less than 0.05 was taken as statistically significant.

## Results

Among 242 students, 65 (26.86%) said that they were diagnosed with a headache disorder by a Neurologist or the General Physician. Out of the diagnosed ones, 45 (69.23%), 14 (21.54%) and 2 (3.08%) had tension, migraine and cluster headache respectively. Four (6.15%) of them had other types of headaches that included secondary causes like the common cold, sinusitis, and refractive error.

“Table 1” shows the prevalence of headaches across socio-demographic variables like age, gender, marital status and MBBS academic years. The median age was similar in both groups; having and not having diagnosed headache disorder (21.00 [20.00–23.00]). Out of the total, 175 (72.31%) were males and 67 (27.69%) were females. The headache was more prevalent in females (20, 29.85%) compared to males (45, 25.71%). The prevalence of headaches was highest among fourth-year students (14/37, 37.84%) followed by first-year students (21/63, 33.33%). But these were not statistically significant.

**Table 1:** Prevalence of headache across different socio-demographic variables.

SN	Variables	Headache		p-value
		Yes	No	
1	Age	21.00 (20.00–23.00)	21.00 (20.00–23.00)	0.896
2	Gender			0.516
	Males	45 (25.71%)	130 (74.29%)	
	Females	20 (29.85%)	47 (70.15%)	
3	Marital status			1.000
	Married	1 (33.33%)	2 (66.67%)	
	Unmarried	64 (26.78%)	175 (73.22%)	
4	Academic years			0.189
	First	21 (33.33%)	42 (66.67%)	
	Second	13 (22.41%)	45 (77.59%)	
	Third	9 (19.15%)	38 (80.85%)	
	Fourth	14 (37.84%)	23 (62.16%)	
	Fifth	8 (21.62%)	29 (78.38%)	

Notes: p-values have been obtained by performing the Mann-Whitney U test and Chi-square/Fisher’s Exact test for continuous and categorical variables respectively.

### Aetiology and precipitating factors

21 (32.31%) of them reported that the headache starts after an illness or infection or accident. The rest of the others had spontaneous onset of headache. ‘Anxiety/stress’ (49/65, 75.40%) was the most common precipitating factor followed by ‘too little sleep’ (30, 46.15%) and ‘loud noise’ (28, 43.07%). Other precipitants of headache are illustrated in “Table 2”.

**Table 2:** Precipitating factors, characteristics and severity of headache.

SN	Variables	n (%)
1	Precipitating factors:	
	• Anxiety/Stress	49 (75.38)
	• Too little sleep	30 (46.15)
	• Loud noise	28 (43.08)
	• Fatigue	25 (38.46)
	• Bright light	23 (35.38)
	• Odours	22 (33.85)
	• Family problems	21 (32.31)
	• Too much sleep	14 (21.54)
2	Timing of headache:	
	• Afternoon	23 (35.38)
	• Night	21 (32.31)
	• Morning	7 (10.77)
	• Afternoon and Night	6 (9.23)
	• Morning, Afternoon and Night	5 (7.69)
	• Morning and Afternoon	2 (3.08)
	• Morning and night	1 (1.54)
3	Location of pain:	
	• Forehead	19 (29.23)
	• All around the head	16 (24.62)
	• Back of head	7 (10.77)
	• Left side	7 (10.77)
	• Top of head	6 (9.23)
	• Temples	5 (7.69)
	• Right side	4 (6.15)
	• Neck	1 (1.54)
4	Character of pain	
	• Dull	17 (26.15)
	• Throbbing/Pounding	12 (18.46)
	• Aching/Pressure	11 (16.92)
	• Tightness like a rubber band wrapped around the head	9 (13.85)
	• Exploding	3 (4.62)
5	Accompanying symptoms	
	• Sensitivity to light	23 (35.38)
	• Nausea	22 (33.85)
	• Confusion	15 (23.07)
	• Weakness of limbs	10 (15.38)
	• Vomiting	9 (13.85)
	• Numbness of limbs	8 (12.31)
	• Sensitivity to odor	8 (12.31)

### Family history of headache

Seven (10.77%) had a positive family history of diagnosed headaches. 54 (83.08%) had no relevant family history. Four (6.15%) of the participants did not give any response to this question.

### Characteristics of headache

The majority of the students (55/65, 84.62%) reported that the headache episodes was intermittent, whereas it was constant in 10 (15.37%) cases. The headache lasts for less than one hour in half of the students (32/65, 49.23%), whereas it lasted for one to two hours, three to four hours and more than four hours in 11 (16.92%), 10 (15.38%) and 12 (18.46%) of them. The onset of headache occurred mostly during the afternoon (23, 35.38%) and night (21, 32.31%). Three fourth of the participants (49, 75.38%) said that the pain starts small and gradually builds up. The headache was located on the forehead and all around the head in 19 (29.23%) and 16 (24.62%) students. 17 (26.15%) and 12 (18.46%) reported pain as ‘dull’ and ‘throbbing/pounding’ types respectively as described in “Table 2”.

### Impact of headache on quality of life

The problem of headaches stopped them from doing their regular activities in 41 (63.08%) students. 23 (35.38%) also missed their work or school due to headaches. Eight of them (12.31%) reported waking up at night due to pain. Half of the students (33, 50.77%) said that the headache episodes are becoming more frequent than before. It was lasting longer than in the past in 21 (32.31%) and became stronger in 11 (16.92%) students. Sensitivity to light (23, 35.38%) followed by nausea (22, 33.85%) and confusion (15, 23.08%) were the commonest accompanying symptoms as shown in “Table 2”.

### Relief and treatment

The headache resolved on its own most of the time in 52 (80.00%) students. In cases of non-resolution of headache, the most commonly used self-treatment was oral medication (35, 53.85%). Out of oral medication, non-steroidal anti-inflammatory drugs (26, 40%) were commonly used.

## Discussion

In our study, the headache was found to be fairly common among medical students, with a prevalence of 26.86%. This is considerably lower than reported in studies by Kurt et al. (41.0%), Ojini et al. (46.0%), Ghorbani et al. (58.7%), and Bakhshi et al. (87.8%), and Anaya et al. (81.8%) [11–15]. The prevalence was higher in females (29.85%) than in males (25.71%). This is consistent with studies were done by Ojini et al. and Bakhshi et al. which showed a higher prevalence rate in females than males [12, 15]. The studies done on the general population have also shown a higher prevalence of headaches in women than men [16, 17]. Tension-type headache was found to be the most common headache with a prevalence of 69.23% which is significantly higher than the global prevalence. However, this value is similar to the findings in several other studies conducted in Ukraine (58.7%), Palestine (59.8%), Nigeria (18.1%), Iran(44.2%), and Nairobi (50%) [11, 12, 15, 18]. Similarly, in our study, tension headache (69.23%) was most common followed by migraine (21.54%). It is in line with the findings of a study conducted in Ukraine (9.1%). The prevalence of migraine headaches in our case is higher as compared to the global prevalence and a study in Ukraine (9.1%) [19, 20].

In our scenario, the prevalence of headaches was lower in third-year MBBS students (19.15%) compared to other academic years. This may be because the students in the third year have to study fewer subjects compared to other academic years as per the curriculum of the university. Fewer subjects and exams, and thus reduced stress can explain this finding. Similar findings have been seen in a study done in Palestine by Anaya et al. which found differences in basic and clinical years [11]. Also, fourth-year students had a higher prevalence of headaches, perhaps owing to multiple subjects that needed to be studied in a year. A study done in Iran by Ghorbani et al. also found a higher prevalence in the third year which was associated with highly stressful Basic Science exams [13].

In our result, 10.77% of those diagnosed with headaches had a positive family history. This is similar to the study done by Ghorbani et al. (10.0%) in Iran but less than that reported in studies by Ojini et al. (22%) and Deleu et al. (57.6%) done in Nigeria and Oman respectively [13, 15, 21]. This could be due to the racial and genetic differences between different populations for which separate population-based studies may be required.

Our study shows that anxiety/stress (75.38%) related to exams was the major precipitating factor for headaches followed by too little sleep (46.15%). This finding is similar to the studies conducted in Ukraine (36%) and Indonesia (20%), which also showed anxiety to be the most common cause of headaches [19, 22]. The major site of headache in our setting was the forehead (29.23%) followed by “all around the head” (24.62%). This finding is different from the study conducted in Saudi Arabia which showed the most common headache location to be bilateral [23]. The major characteristic of headache was found to be the dull type (26.15%) followed by throbbing/pounding in nature (18.46%). This finding also differs from the finding of the study conducted in Indonesia which showed more than half of the cases to be pulsatile and one-third with a sensation of tightness around the headache [22].

Our study showed most of the headache is accompanied by sensitivity to light (35.38%) followed by nausea (33.85%). This finding is comparable to an Indonesian study, which showed nausea (18.1%) to be the most common accompanying symptom [22]. Our study depicted that 63.08% of those having headaches couldn’t perform their regular activities, and about 35.38% were found to miss their work or school. This finding is significantly higher than in Indonesia which showed only 41.5% of the participants to have severe debilitating headaches and around 41.3% to have difficulty in performing daily activities [22].

In our study, 80% of the participants with headaches had spontaneous resolution of symptoms. Among those not having a spontaneous resolution, 53.85% were found to do self-medication. This is much lower than the findings from the study conducted in Oman (80.3%) but higher than the findings of studies conducted in Turkey (47.6%) and Pakistan (41%) [21, 24, 25]. Among those who practised self-medication, non-steroidal anti-inflammatory drug (40%) was found to be the most commonly used medicine. However, a study by Mohamad Shadi Alkarrash et al, depicted that 91.1% of the students used Acetaminophen for self-medication purposes [26]. The reason behind this may be NSAIDs and Acetaminophen being easily available over-the-counter drugs.

There are some limitations of this study to be mentioned. This is a single center study and the results of this study may not be representative. Since it was conducted with a Semi-structured questionnaire distributed through an online platform, there might have been some information bias. Some of the medical students had class tests or presentations at the time of data collection so it could have impacted headache development and characteristics. Also, the students were engaged in online education during the past year due to COVID-19 which itself could be perceived as stressful and overwhelming to some students. In this study, we have considered only diagnosed cases to have a headache disorder, so there might have been missing cases that were not diagnosed yet. These cases need to be examined by the Neurologist or the General Physician. The cause of secondary headaches is also not properly addressed. Also, treatment for headaches is not described in detail as this study is more focused on describing characteristics rather than treatment. So, further studies need to be conducted in future to get the real picture. However, this study is relevant to describing the load and characteristics of headaches in medical students.

## Conclusion

One-fourth of the medical student had headache disorder, with tension headache being the most common type. The fourth- and first-year students were most commonly affected, and anxiety/stress was their commonest precipitant. The problem of headaches stopped the majority of them from doing regular activities. Half of the students reported practising self-medication in case of non-resolution of pain. Therefore, this problem should be properly looked into by the concerned authority and the guardians. Further multicenter studies are required to study the complete clinical characteristics of headaches among medical students in Nepal.

## Supporting information

S1 File (DOCX)
