# Supplementary material for: Prevalence and clinical characteristics of headache among medical students of Nepal: A cross-sectional study
Source: PLoS One. 2022 Nov 18;17(11):e0277821. doi: 10.1371/journal.pone.0277821 (PMC9674122; doi:10.1371/journal.pone.0277821)
Supplement: S1 File — (DOCX) [file pone.0277821.s001.docx]

# Supporting Information

**S1 Data**.

[**https://doi.org/10.6084/m9.figshare.20124026**](https://doi.org/10.6084/m9.figshare.20124026)
